# Preparation of Microencapsulated* Bacillus subtilis* SL-13 Seed Coating Agents and Their Effects on the Growth of Cotton Seedlings

**DOI:** 10.1155/2016/3251357

**Published:** 2016-01-14

**Authors:** Liang Tu, Yanhui He, Chunhui Shan, Zhansheng Wu

**Affiliations:** School of Chemistry and Chemical Engineering, Shihezi University, Shihezi 832003, China

## Abstract

Inoculation of the bacterial cells of microbial seed coating agents (SCAs) into the environment may result in limited survival and colonization. Therefore, the application efficacy of an encapsulated microbial seed coating agent (ESCA) was investigated on potted cotton plants; the agent was prepared with polyvinyl alcohol, sodium dodecyl sulfate, bentonite, and microencapsulated* Bacillus subtilis* SL-13. Scanning electron micrography revealed that the microcapsules were attached to ESCA membranes. The ESCA film was uniform, bubble-free, and easy to peel. The bacterial contents of seeds coated with each ESCA treatment reached 10^6^ cfu/seed. Results indicated that the germination rate of cotton seeds treated with ESCA_4_ (1.0% (w/v) sodium alginate, 4.0% polyvinyl alcohol, 1.0% sodium dodecyl sulfate, 0.6% acacia, 0.5% bentonite, and 10% (v/v) microcapsules) increased by 28.74%. Other growth factors of the cotton seedlings, such as plant height, root length, whole plant fresh weight, and whole plant dry weight, increased by 52.70%, 25.13%, 46.47%, and 33.21%, respectively. Further analysis demonstrated that the peroxidase and superoxide dismutase activities of cotton seedlings improved, whereas their malondialdehyde contents decreased. Therefore, the ESCA can efficiently improve seed germination, root length, and growth. The proposed ESCA exhibits great potential as an alternative to traditional SCA in future agricultural applications.

## 1. Introduction

Viable seeds form the basis of crop production [1]. Seed research has focused on providing technology that can sustain or increase the viability and health of successful germination [2, 3]. Seed coating technologies using various treatment methods have been developed to cope with various constraints, such as drought and hunger, seedling diseases, seed dormancy, or suboptimal temperatures. These techniques have been widely used for crop protection and prevention of massive agricultural loss [4]. Seed coating agents (SCAs) are often formulated from aqueous suspension preparations by using the original drug (e.g., pesticide, bactericide, microorganism, and fertilizer), membrane formers, dispersants, thickeners, and other additives.

At present, chemical and biological SCAs are widely used in agriculture to control pests and diseases. However, the majority of chemical SCAs fail to provide efficient alternatives for the environment because of their cumulative toxicity [5, 6]. Biological SCA, an environmentally friendly agent, contains bacteria or metabolites as main active ingredients to improve plant resistance. Previous studies have indicated that biological SCA can be used to coat seeds and improve seed germination, seedling length, root length, and plant growth [7–9]. Wu et al. [10] reported that the germination rate of cotton seeds treated with biological SCA containing* Klebsiella oxytoca *Rs-5 and* Bacillus subtilis* increased by 11.3%. Angelopoulou et al. [11] demonstrated that two biological SCAs containing* Paenibacillus alvei* K165 or the nonpathogenic* Fusarium oxysporum* F2 protected plants against* Verticillium dahliae* and promoted plant growth.

In our previous studies,* B. subtilis* SL-13 producing chitinase activity was obtained from cotton field soil in Xinjiang province and proven to promote sprouting and seedling growth in tomatoes [12]. However, inoculation of the cells of the SCA into the environment may result in poor survival and colonization because of their susceptibility to various environmental factors. Encapsulation of bacteria can effectively protect sensitive bacteria from unfavorable environments [13]. In addition, microcapsules could be designed to gradually or in a controlled manner deliver core materials by bursting, diffusion, and dissolution. SCAs may enhance the survival of cells and protect the microorganisms against numerous environmental stresses. Developing an encapsulated microbial seed coating agent (ESCA) with encapsulated* B. subtilis* SL-13 showing desirable properties, such as the ability to improve seed germination, and meeting various agricultural needs is a promising endeavor. Currently, methods to prepare and apply ESCAs remain poorly investigated. Therefore, studies on ESCAs are innovative, important, and worthy of investigation.

This study aimed to propose a method of preparing an ESCA with sodium alginate, polyvinyl alcohol (PVA), sodium dodecyl sulfate, and bentonite. We evaluated the properties of the ESCA, including its film-forming time, degree of uniformity, shedding rate, and suspension rate, and application efficacy in cotton. Cotton seed germination rates, cotton biomass, and peroxidase (POD), superoxide dismutase (SOD), and malondialdehyde (MDA) contents of cotton seedlings, among other effects, were also evaluated. The results of this study can provide significant theoretical basis and technical support for the production and application of ESCAs.

## 2. Materials and Methods

### 2.1. Materials

Sodium alginate and bentonite were purchased from Xilong Chemical Co., Ltd. (Guangdong, China). Sodium dodecyl sulfate was provided from Fuchen Chemical Co.; acacia was purchased from Hengxing Chemical Co., Ltd. (Tianjin, China). All chemicals and reagents used were of analytical grade. The strain* Bacillus subtilis* SL-13 used in this study was previously isolated from rhizosphere in Xinjiang province of China [12]. The* B. subtilis* SL-13 were cultured in nutrient agar (NA) liquid medium (5 g beef extract, 10 g peptone, 5 g NaCl, 1000 mL H_2_O, pH 7.0–7.2) with shaking at 200 rpm and 30°C for 36 h. The cells were harvested at stationary phase and determined as 10^13^ colony-forming units (cfu)/mL of concentration in NA agar plates for overnight incubation at 30°C by spread plate method.

### 2.2. Preparation of Microparticles

*B. subtilis* SL-13 microparticle preparation was conducted similarly to the encapsulation procedure described in our previous study [14]. The microparticles collected exhibited an encapsulation efficiency of 93.44%. The emulsion was composed of an upper oil phase and a water phase. The water phase containing microcapsules was preserved to prepare ESCAs at 4°C.

### 2.3. Preparation of ESCA

Sodium alginate and PVA were dissolved in water to form a film-forming agent. Other additives, including dispersants (sodium dodecyl sulfate), thickeners (acacia), and fillers (bentonite), were added and uniformly dissolved into the film-forming agent. A water emulsion (10 mL) containing about 1.03 g of wet microcapsules was added, and the mixture was stirred in an electronic constant-speed mixer for several minutes to finish preparing the ESCAs. The relevant formulas are listed in Table 1.

### 2.4. Characterization of ESCA 

#### 2.4.1. Scanning Electron Micrograph (SEM) Analysis

Samples were dried at 50°C for 48 h before being coated with gold for SEM analysis. The morphology of the microcapsules was analyzed by using a SEM (JSM-6700F, Jeol, Tokyo, Japan).

#### 2.4.2. Film-Forming Time

A stable membrane was formed on a dry glass plate and then placed in an oven at 35°C to dry. The time spent to complete this step was recorded.

#### 2.4.3. Degree of Uniformity, Shedding Rate, and Suspension Rate

The degree of uniformity and shedding rate were determined according to Standard Testing Methods of China GB/T1 7768-1999, and the suspension rate of the SCA was investigated by the Standard Testing Methods of China GB/T1 14825-2006, as described by Honglu and Guomei [15].

#### 2.4.4. Swelling Analysis

Dried ESCA membranes (1 g) peeled from the dry glass plate were placed in a 10 mL EP tube to which distilled water was subsequently added. The membranes were taken out of the tube after immersion for 4 h, and excess water was removed using tissue paper.

#### 2.4.5. Coating Amount of Functional Bacteria

The cotton seeds were sterilized by immersion in 70% ethanol for 5 min and subsequently in 0.1% HgCl_2_ for 10 min and washed several times with sterile water. Sterilized cotton seeds obtained were coated with the ESCA at a proportion of 20 : 1 (w/v) and then dried by airing for 20 min. The dried coating film in a certain number of seeds was stripped down, added to 10 mL of sterile physiological saline (0.9%), and ground. Viable bacteria were determined by counting the colony-forming units (cfu) present on NA agar plates after serial dilution-incubation at 30°C for 48 h. The coating amount of functional bacteria in a cotton seed was calculated.

### 2.5. Pot Experiments

Pot experiments of the cotton plants were set up with five formations of seed coating: ESCA_2_, ESCA_4_, ESCA_8_, bacteria-free seed coating agent (SCA_F_), and no-treatment control (NC). Ten cotton seeds were sown in each pot filled with 100 g of vermiculite (diameter 0.5 mm); each treatment included eight replicates. The pots were arranged in a randomized design, placed in an artificial climatic box with a day/night temperature at 20°C, supplied with 400 *μ*mol photons (m^2^s)^−1^ of light for 14 h during the day, and maintained at 60% humidity by irrigation with sterile water using injection 4-5 times a week.

#### 2.5.1. Growth Parameters

The germination rate was determined to be 10 d after planting. After sowing for 50 d, 3 plants from each replicate were randomly harvested, and data on plant growth variables were recorded. These variables included germination, plant height, root length, whole plant fresh weight, and whole plant dry weight. The plant material for whole plant dry weight determination was dried at 105°C for 10 min and then at 75°C to obtain a constant weight.

#### 2.5.2. Determination of POD Activity, SOD Activity, and MDA Content of Cotton Seedlings

About 0.2 g of fresh leaves samples was ground to powder in liquid nitrogen and then homogenized with 1 mL of 0.05 mol·L^−1^ phosphate buffer solution (pH 7.8). The extracts were centrifuged at 8,500 rpm for 20 min at 4°C, and the supernates were collected for antioxidant enzyme activity assay. The MDA content of the cotton seedlings was measured using the thiobarbituric acid method, as described by Zhao et al. [16]. Specific absorbance at 532 nm and nonspecific absorbance at 600 nm were also measured. SOD activity was detected using the NBT method [17], and POD activity was measured in accordance with the guaiacol method described by Wang et al. [18]. All measurements were repeated thrice for each treatment.

### 2.6. Statistical Analysis and Control

The results were expressed as the mean and standard deviation of triplicate studies. Statistical differences were tested with one-way ANOVA with Tukey's multiple comparison tests. Any *p* values less than 0.05 were considered to be significant.

## 3. Results and Discussion

### 3.1. SEM Analysis

**Figure 1:**
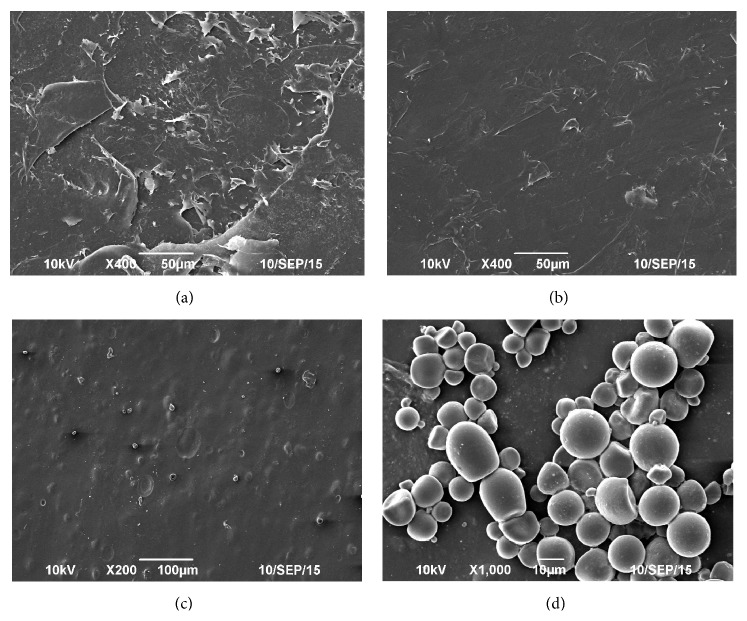
Scanning electron micrographs of the PVA-alginate film without microcapsules (a), the PVA-alginate-bentonite film without microcapsules (b), and the PVA-alginate-bentonite film with microcapsules (c and d).

SEM micrographs reveal the structure of the SCA film (Figure 1). The PVA-alginate film was irregular and presented cracks and faults, as shown in Figure 1(a). The PVA-alginate-bentonite film was more regular than the PVA-alginate film (Figure 1(b)). The observed changes could be attributed to intermolecular hydrogen bonding between silanol groups (Si−OH) on the bentonite surface and the hydroxyl or carboxyl groups of alginate and PVA [19]. Homogeneous films favor drug release and seed protection. SEM showed microcapsules exhibiting a spherical shapes, smooth surfaces, and good dispersion (Figure 1(d)). The particle size of the microcapsules ranged from 5 *µ*m to 15 *µ*m, and the microcapsules were attached to the ESCA films, thereby demonstrating the feasibility of the ESCA (Figures 1(c) and 1(d)) [20]. Therefore, the proposed technique provides a novel method for developing an SCA.

### 3.2. Effect of Varying Composition Proportions on ESCA

The suspension rates of different matrices are listed in Table 2. The suspension rate was enhanced as the dispersant (sodium dodecyl sulfate) content increased; this phenomenon may be due to the improved dispersibility of the respective components and reductions in aggregation. The bentonite structure presented a high specific surface area and ion change capability, resulting in high adsorption [15]. When bentonite was added to the complex system, the suspension rates of all samples improved by adsorption. The maximum suspension rate of ESCA was 99.53%, and the film-forming time was less than 15 min. The ESCA film was uniform, bubble-free, and easy to peel.

As indicated in Table 2, the swelling ratio also improved as PVA concentration increased. This increase in swelling ratio may be due to increases in the number of inter-/intramolecular connections formed by the reaction of alginate with available sites in PVA containing large amounts of –OH and –COO^−^ and absorbed water [21]. Besides ESCA_1_, all other samples exhibited a degree of uniformity exceeding 90% and shedding rates less than 1%. Thus, the improved uniformity and reduced shedding rate afforded by ESCA reveal its potential for wider utilization to achieve reduced drug loss.

### 3.3. Coating Bacterial Amount of ESCA 

**Figure 2:**
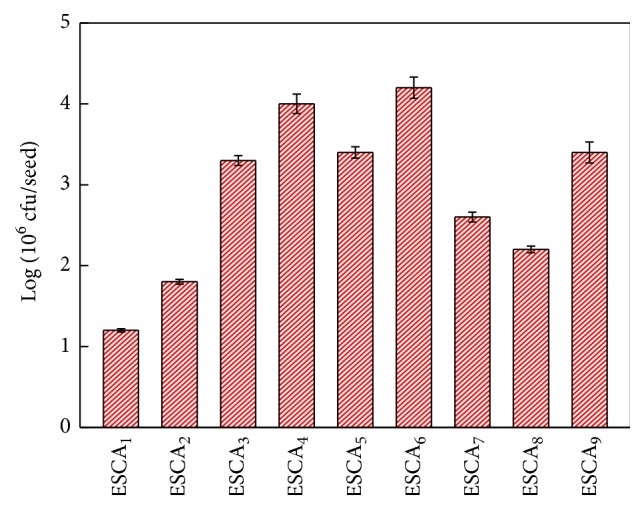
Coating amount of functional bacteria on cotton seeds.

The coating bacterial amounts of different ESCAs in the cotton seeds are shown in Figure 2. The coating bacterial contents of each ESCA reached 10^6^ cfu/seed, with the maximum coating bacterial amount of ESCA_6_ being 6.42 × 10^6^ cfu/seed.* B. subtilis* SL-13 was kept at a higher order of magnitude and promoted seed germination and seedling growth. Moreover, the microcapsules delayed the survival time and release of cells in a controlled manner, keeping the bacterial content of the ESCAs adequately high for enhanced utilization and extending the utilization period [14].

### 3.4. Growth Parameters

The effects of applying* B. subtilis* SL-13 microcapsule-based SCA on the germination rate of cotton seeds and growth of cotton seedlings were evaluated (Table 3). The germination rate in the NC treatment group was found to be 46.58%; in comparison to this group, the germination rates of the SCA_F_, ESCA_2_, ESCA_4_, and ESCA_8_ groups increased by 11.86%, 19.09%, 28.74%, and 24.06%, respectively. All ESCA treatments showed germination rates higher than that of the SCA_F_ treatment, which may be attributed to the ability of microcapsules to protect microorganisms against environmental stresses, thereby allowing a large quantity of bacteria to be stably released from the microcapsules and colonized in the cotton seeds.

The results indicated that ESCA treatments could significantly (*p* ≤ 0.05) increase the cotton biomass compared with the control plants (Table 3). After a planting period of 50 d, the plant height of the untreated cotton seedlings was 11.48 cm; when treated with SCA_F_, the plant height of the cotton seedlings increased by 15.77%. Plant heights also significantly increased by 41.46%, 52.70%, and 44.43% when treated with ESCA_2_, ESCA_4_, and ESCA_8_, respectively. The root length, whole plant fresh weight, and whole plant dry weight of plants treated with SCA_F_ and ESCA yielded similar results. These effects on cotton seedlings may be due to the action of* B. subtilis* as an inducer of various phytohormones, such as indoleacetic acid, abscisic acid, organic acid, gibberellins, and cytokinins. These phytohormones favor root growth and increase the number of root hairs [22]. In addition, seeds coated with* B. subtilis* SL-13 ESCA exhibited seed germination and biomass values superior to those of seeds treated with SCA_F_. This result could be attributed to the improved abundance of bacteria released from ESCA in the rhizosphere of the cotton plants. Thus, ESCA can be applied as a biotechnological agent to improve the growth of plant seedlings.

### 3.5. Effect of ESCA on MDA Content of Cotton Seedlings

**Figure 3:**
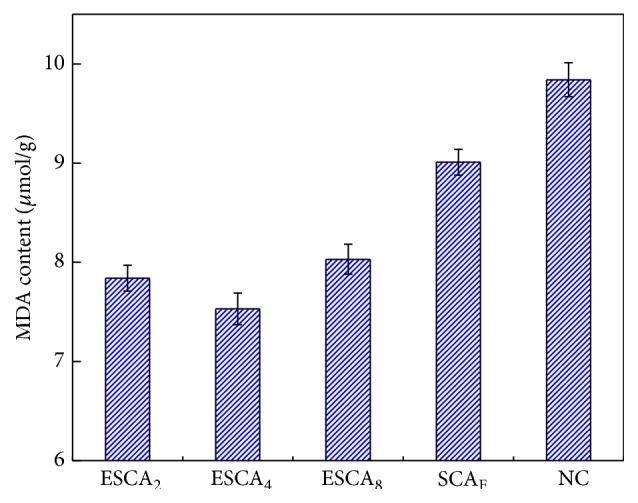
Effect of different treatments on the MDA concentration of cotton seedlings. Error bars indicate the standard error of means (±SE).

MDA, a measure of lipid peroxidation, decreased in all ESCA treatments, in contrast to observations in cotton seedlings treated with NC and SCA_F_ (Figure 3). The MDA content in NC was 9.48 *μ*mol/g; compared with this group, MDA contents in the SCA_F_, ESCA_2_, ESCA_4_, and ESCA_8_ groups decreased by 8.53%, 20.32%, 23.48%, and 18.39%, respectively. This decrease in MDA content indicates that the ESCA can reduce the membrane lipid peroxidation of cotton seedlings effectively, thus promoting the root growth and seedling growth [23].

### 3.6. Effect of ESCA on Enzymatic Activities of Cotton Seedlings

**Figure 4:**
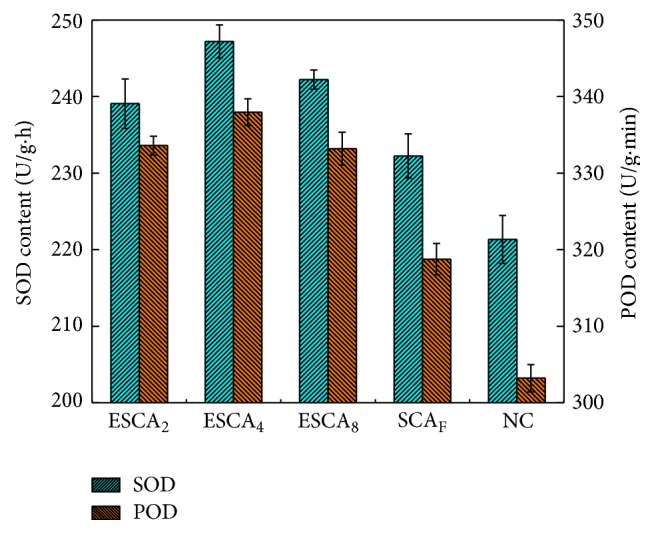
Effects of different seed coating agents on SOD and POD content.

SOD and POD contents were affected by the different ESCA treatments, as shown in Figure 4. A significant (*p* ≤ 0.05) increase in the SOD and POD contents of the cotton plant leaves was observed after ESCA treatments at specific monitoring times. The SOD and POD contents of the plants in NC were 221.34 and 303.21 U/g·min, respectively. The SOD content of the plants treated with SCA_F_, ESCA_2_, ESCA_4_, and ESCA_8_ increased by 5.06%, 8.01%, 11.68%, and 9.44%, respectively, relative to the control group. The POD content of plants treated with SCA_F_ increased by 5.13% compared with that of NC. Treatment with ESCA_2_, ESCA_4_, and ESCA_8_ enhanced POD contents by 10.02%, 11.46%, and 9.89%, respectively, relative to NC. This increase in POD and SOD contents indicates that the ESCA can improve the seedling radical scavenging related enzyme activity and the content of radical scavenger, thus promoting the root growth and seedling growth [23]. Increases in the POD and SOD activities of cotton seedlings treated with ESCA can promote the metabolism of the cottons and increased the seed germination rate and biomass through the role of the enzyme.

## 4. Conclusions

This study demonstrated a feasible method to prepare ESCA as an alternative to traditional SCAs. Overall, the results indicated that sodium dodecyl sulfate, acacia, and bentonite could be potentially applied to enhance the swelling analysis, degree of uniformity, and suspension rate of* B. subtilis *SL-13 microcapsule-based SCA. SEM revealed that the microcapsules were attached to the ESCA membranes. The uniformity of a vast majority of samples exceeded 90%, and the shedding rate of all samples was less than 1%. The coating bacterial content of each ESCA reached 10^6^ cfu/seed. Pot results showed that the ESCA_2_, ESCA_4_, and ESCA_8_ treatments significantly improved the cotton growth by increasing germination rate, plant height, whole plant fresh weight, whole plant dry weight, and shoot length of the plants. Moreover, the POD and SOD activities of the ESCA-treated cotton seedlings improved, and their MDA contents decreased. This study provides valuable information regarding the practical application of encapsulated microbial SCAs in farmlands.

## Figures and Tables

**Table 1:** The composition and ratio of ESCA.

SCA types	Membrane formers (w/v%)		Dispersant (w/v%)	Thickeners (w/v%)	Bentonite (w/v%)	Microcapsules (v/v%)
	Alginate	PVA				
ESCA_1_	1.0	3.0	1.0	0.3	0.5	10
ESCA_2_	1.0	3.0	2.0	0.6	0.5	10
ESCA_3_	1.0	3.0	3.0	0.9	0.5	10
ESCA_4_	1.0	4.0	1.0	0.6	0.5	10
ESCA_5_	1.0	4.0	2.0	0.9	0.5	10
ESCA_6_	1.0	4.0	3.0	0.3	0.5	10
ESCA_7_	1.0	5.0	1.0	0.9	0.5	10
ESCA_8_	1.0	5.0	2.0	0.3	0.5	10
ESCA_9_	1.0	5.0	3.0	0.6	0.5	10

**Table 2:** Properties of ESCA.

Samples	Suspension rate (%)	Film-forming time (min)	Film-forming properties	Swelling ratio (%)	Uniformity (%)	Shedding rate (%)
ESCA_1_	97.02 ± 1.23	8 ± 0.02	0	249.78 ± 4.21	86.12 ± 3.12	0.26 ± 0.008
ESCA_2_	98.10 ± 2.31	9 ± 0.03	+	213.24 ± 3.68	90.64 ± 2.87	0.32 ± 0.012
ESCA_3_	99.62 ± 2.17	10 ± 0.03	+	182.06 ± 2.56	95.25 ± 3.18	0.41 ± 0.017
ESCA_4_	97.35 ± 1.09	8 ± 0.01	+	317.47 ± 3.14	95.57 ± 2.96	0.20 ± 0.004
ESCA_5_	99.24 ± 2.58	10 ± 0.02	+	259.99 ± 2.17	95.46 ± 4.12	0.48 ± 0.019
ESCA_6_	99.43 ± 1.74	12 ± 0.04	+	225.36 ± 1.38	93.28 ± 3.35	0.52 ± 0.023
ESCA_7_	97.83 ± 0.86	15 ± 0.03	0	278.58 ± 2.53	90.65 ± 2.16	0.27 ± 0.006
ESCA_8_	98.60 ± 1.26	9 ± 0.04	+	454.98 ± 5.86	94.26 ± 1.97	0.43 ± 0.015
ESCA_9_	99.53 ± 3.12	12 ± 0.03	+	279.80 ± 3.15	95.34 ± 2.84	0.62 ± 0.024

Notes: mean ± standard errors. Significant differences were determined according to Student's *t*-test with *p* ≤ 0.05.

+ denotes a uniform, bubble-free, and easy to peel film formed on the glass plate; 0 represents an uneven film formed on the glass plate.

**Table 3:** Effects of ESCA on germination rate and seedling biomass.

Treatments	Germination rate (%)	Plant height (cm)	Root length (cm)	Whole plant fresh weight (g)	Whole plant dry weight (g)
NC	46.58 ± 1.34	11.48 ± 0.51	5.30 ± 0.12	2.13 ± 0.038	1.32 ± 0.006
SCA_F_	58.44 ± 3.48	13.29 ± 0.45	5.84 ± 0.38	2.62 ± 0.045	1.40 ± 0.018
ESCA_2_	65.67 ± 2.24	16.24 ± 0.63	6.12 ± 0.27	2.65 ± 0.061	1.53 ± 0.015
ESCA_4_	75.32 ± 1.94	17.53 ± 0.48	7.06 ± 0.35	3.12 ± 0.076	1.66 ± 0.023
ESCA_8_	70.64 ± 3.82	16.58 ± 0.53	6.48 ± 0.34	2.98 ± 0.075	1.61 ± 0.015

Notes: error bars indicate the standard error of means (±SE).
